# Monitoring Interest in Herpes Zoster Vaccination: Analysis of Google Search Data

**DOI:** 10.2196/10180

**Published:** 2018-05-02

**Authors:** Elyse J Berlinberg, Michael S Deiner, Travis C Porco, Nisha R Acharya

**Affiliations:** ^1^ Francis I Proctor Foundation University of California, San Francisco San Francisco, CA United States; ^2^ Department of Ophthalmology University of California, San Francisco San Francisco, CA United States; ^3^ Department of Epidemiology & Biostatistics University of California, San Francisco San Francisco, CA United States; ^4^ Institute for Global Health Sciences University of California, San Francisco San Francisco, CA United States

**Keywords:** herpes zoster, vaccination, Internet, periodicity, Google Trends, infodemiology

## Abstract

**Background:**

A new recombinant subunit vaccine for herpes zoster (HZ or shingles) was approved by the United States Food and Drug Administration on October 20, 2017 and is expected to replace the previous live attenuated vaccine. There have been low coverage rates with the live attenuated vaccine (Zostavax), ranging from 12-32% of eligible patients receiving the HZ vaccine.

**Objective:**

This study aimed to provide insight into trends and potential reasons for interest in HZ vaccination.

**Methods:**

Internet search data were queried from the Google Health application programming interface from 2004-2017. Seasonality of normalized search volume was analyzed using wavelets and Fisher’s g test.

**Results:**

The search terms “shingles vaccine,” “zoster vaccine,” and “zostavax” all exhibited significant periodicity in the fall months (*P*<.001), with sharp increases after recommendations for vaccination by public health-related organizations. Although the terms “shingles blisters,” “shingles itch,” “shingles rash,” “skin rash,” and “shingles medicine” exhibited statistically significant periodicities with a seasonal peak in the summer (*P*<.001), the terms “shingles contagious,” “shingles pain,” “shingles treatment,” and “shingles symptoms” did not reveal an annual trend.

**Conclusions:**

There may be increased interest in HZ vaccination during the fall and after public health organization recommendations are broadcast. This finding points to the possibility that increased awareness of the vaccine through public health announcements could be evaluated as a potential intervention for increasing vaccine coverage.

## Introduction

One in three Americans will experience shingles (herpes zoster; HZ) in their lifetime, which may lead to extremely painful postherpetic neuralgia [1]. HZ of the eye, or herpes zoster ophthalmicus, is the second most common location of HZ and can lead to blindness [1-4]. On October 20, 2017, the United States Food and Drug Administration (FDA) approved Shingrix (GlaxoSmithKline), a new recombinant subunit vaccine for HZ [5]. The recombinant subunit vaccine has been shown to have over 95% efficacy in clinical trials and is now Centers for Disease Control and Prevention (CDC)-preferred over the live attenuated vaccine Zostavax (Merck) released in 2006 [6,7]. However, many lessons can be learned from this first experience with HZ vaccination. Despite showing over 51% and 70% efficacy in adults over age 50 and 60, respectively, the live attenuated vaccine had low coverage rates: studies estimate between 12% and 32% of vaccine-eligible adults have received the HZ vaccine [8-10]. Many possible contributing factors to the low vaccination rate have been proposed, including high cost, a lack of consensus on vaccine recommendation, waning efficacy, supply issues, and general lack of awareness of the vaccine and its benefits [11-20]. Influences on general awareness and interest in the HZ vaccine are poorly understood, and if known they could be utilized in designing more effective public health efforts to increase HZ immunizations. We sought to identify some of the factors affecting public interest in HZ and its vaccine.

Over the past several years, analysis of social media and search data has been of increasing interest in public health efforts [21,22]. This approach has been used to estimate disease incidence, identify and predict potential epidemic outbreaks, assess general understanding of a disease, and gauge public interest in a health-related topic [23-33]. In this study, we analyzed Google Search data to assess past trends in public interest for HZ vaccination in an attempt to better understand what factors may impact HZ vaccine utilization. These findings can inform possible future interventions that could help increase HZ vaccine awareness and uptake for eligible patients.

## Methods

Search data were obtained from the Google Health application programming interface (API; Alphabet, Inc) using specific search terms related to HZ or HZ vaccination (“shingles vaccine,” “zoster vaccine,” “Zostavax,” “medicare vaccine,” “shingrix,” “shingles blisters,” “shingles itch,” “shingles rash,” “skin rash,” “shingles medicine,” “eye shingles,” “shingles contagious,” “shingles pain,” “shingles treatment,” and “shingles symptoms”) and negative control search terms (“roof shingle,” “roof shingles,” “roof repair,” and “roof symptoms”). Unless otherwise specified, all API queries limited results to searches made with a United States geolocation. For each term, the Google Health API was queried for all search data between January 1, 2004 and December 1, 2017. Weekly 2017 data were obtained from the public Google Trends site, which automatically normalizes searches as a relative volume, reported as a number between 0 and 1. Google API data is presented in a grouped fashion to protect privacy per Google API data user requirements.

We assessed periodicity in searches using the weekly or monthly relative intensity of the search data queried between January 1, 2011 and December 31, 2016. Significance of periodicity was tested using Fisher’s g test [34]. To compare seasonal timing between different search terms, we fit trigonometric regression models. We used the equation:

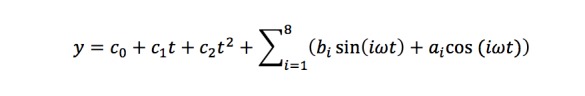



 and used time series bootstrap [35] to test the null hypothesis that the difference in the phase angle arctan
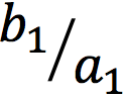
 for the annual terms was zero. A fixed width of 4 was chosen for bootstrap resampling of residuals, with sensitivity analysis using a width of 2 and 8.

Time-frequency analysis was conducted using Morlet wavelets [36], which were estimated using the WaveletComp package in R. A *P*-value of less than .05 was considered significant. All statistical tests were conducted in R version 3.3 for MacIntosh (The R Foundation for Statistical Computing, Vienna, Austria).

## Results

### Seasonal Patterns of Searches Related to Herpes Zoster Vaccination

**Figure 1:**
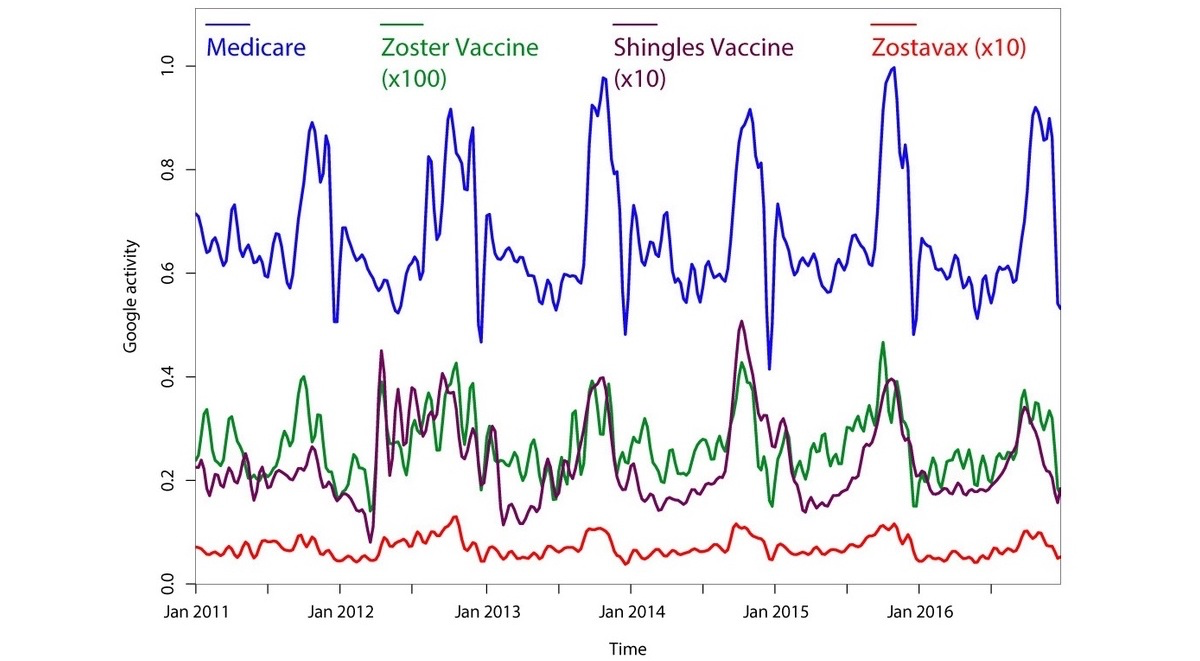
Smoothed Google searches for herpes zoster vaccination-related terms from 2011-2017, normalized as relative search volume (0-1.0). Smoothing was conducted using Morlet wavelet denoising (lower period of 4 weeks, upper period of 128 weeks, omitting terms nonsignificant at the P=.05 level).

Google Health API was queried for all searches related to HZ vaccination between January 1, 2011 and December 31, 2016. The relative frequency of searches for the terms “shingles vaccine,” “zoster vaccine,” and “zostavax” are shown in Figure 1. Trend lines for these terms exhibited a significant annual periodicity with a maximum in the fall (*P*<.001 by Fisher’s g test).

Our initial hypothesis on the possible cause of this seasonal trend is that it is related to insurance utilization. Since deductibles are generally reset on January 1^st^ in American health insurance systems, it could be that more HZ vaccine-eligible patients would seek out this elective immunization in the last few months of the year. Furthermore, the majority of patients receiving the HZ vaccine are Medicare-eligible. The open enrollment period for Medicare Advantage and the Medicare Drug Plan runs between mid-October and December [37]. Newly eligible patients may look online for ways to apply their new insurance policy immediately after they enroll. To test this hypothesis, we assessed the seasonality of searches for “medicare” (Figure 1, blue) and “medicare vaccine” (data not shown). These terms revealed a similar annual cycle as “shingles vaccine,” “zoster vaccine,” and “Zostavax,” with a significant seasonal trend peaking in the fall (*P*<.001 for both terms, Fisher’s g test).

In addition, it has been shown that covaccination for influenza and HZ is safe [38]. One could also hypothesize that the surge in interest for the HZ vaccine may be linked to the flu season. We assessed periodicity for searches for “flu vaccine” and found that it also exhibited a pronounced annual cycle (*P*<.001, Fisher’s g test) with a peak in the fall (data not shown).

We also considered that the seasonal pattern may be related to commercial advertising for the vaccine. Merck produced two widely viewed commercials featuring a popular former National Football League quarterback, initially released in September 2014 and 2015 [39,40]. These commercials may have led individuals to then search online for more information about the vaccine. However, this does not provide a complete explanation of the seasonal trend; Merck has also released other shingles-related commercials in February, April, May, June, July, and August.

### Significant Events in Herpes Zoster Vaccination

**Figure 2:**
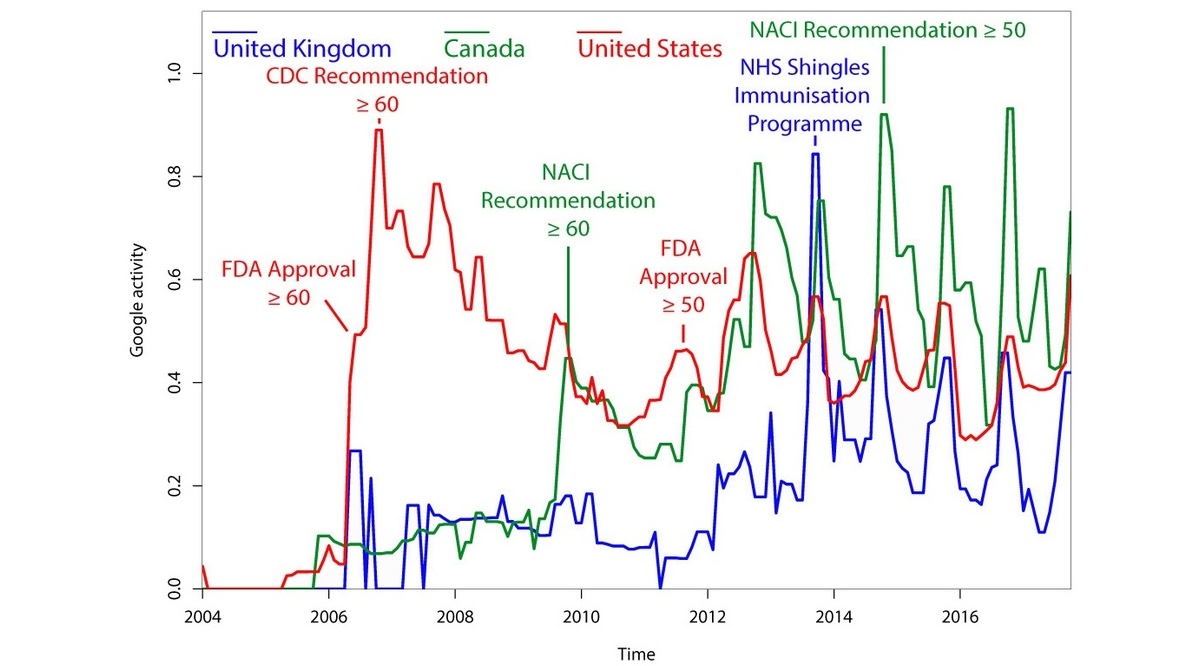
Trends in Google searches for herpes zoster vaccination-related terms from 2004-2017 for the United States, Canada, and the United Kingdom, shown using 3-point median smooth. Trends are annotated for key dates in live attenuated vaccine recommendation.

In our analysis for a seasonal trend in HZ vaccination, we noted that there were certain time points at which the overall volume of searches for vaccination-related topics sharply increased. We inferred that these time points may correspond with key dates in the development, approval, and extension of the HZ vaccine. The FDA approved the live attenuated vaccine (Zostavax) for US patients aged 60 or older on May 25, 2006 and extended its recommendation to patients aged 50 or older on March 25, 2011 [41,42]. Furthermore, the CDC issued its recommendation for the live attenuated vaccine in adults aged 60 or older on October 25, 2006 [43]. Google Trends data were used to estimate US public interest in the vaccine before and after these key dates (Figure 2, red). Between April 2006 and May 2006, Google searches for the term “zostavax” increased 16-fold. Google searches for “zostavax” reached a maximum in November 2006, shortly after the CDC’s recommendation was broadcast. Google searches for “zostavax” decreased over time after the FDA and CDC recommendations and increased again around the date of the FDA extension in March 2011, when searches increased 1.5-fold over the subsequent 6 months and 2-fold in the following year.

The trend in searches for “zostavax” in the United States was compared to the trend in Canada, where the National Advisory Committee on Immunization (NACI) first recommended the live attenuated vaccine for patients 60 years of age or older in January 2010, and then extended this recommendation to patients 50 years of age or older in January 2014 [44,45]. In Canada (Figure 2, green), searches for “zostavax” increased 2-fold just before the initial offering of the vaccine in 2010, but their maximum did not occur until 2014, when the NACI extended its recommendation to people between 50-59 years of age. US searches for “zostavax” were also compared to searches in the United Kingdom, where National Health Service England first offered the live attenuated vaccine through its *Shingles Immunisation Programme* in September 2013 [46]. Google searches for “zostavax” in the United Kingdom (Figure 2, blue) reached a maximum in October 2013.

**Figure 3:**
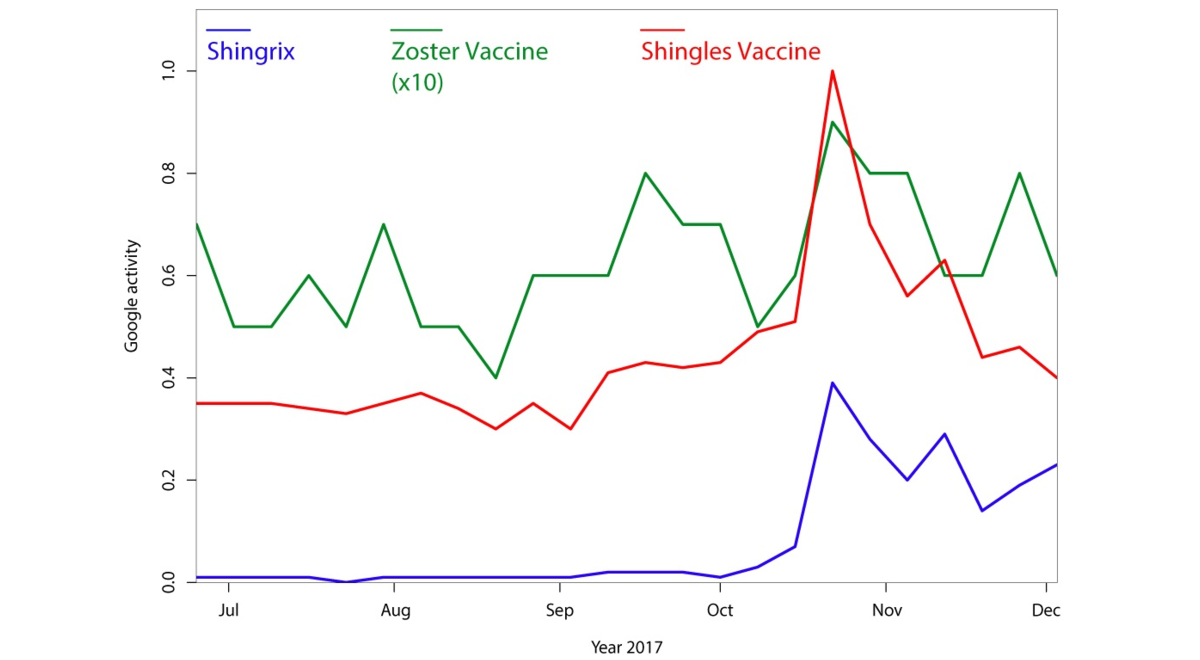
Google search data for herpes zoster vaccination-related terms and the term “shingrix” around its United States Food and Drug Administration approval on October 20, 2017. The 2017 weekly data used for this figure was retrieved from the public Google Trends website, not the Google Health application programming interface.

The FDA approved the new recombinant subunit vaccine (Shingrix) on October 20, 2017 [5]. In light of this recent event, we additionally analyzed Google searches before and after this approval. The results can be found in Figure 3. After FDA approval, Google searches for the term “shingrix” increased over 6-fold with an accompanying 2-fold increase in searches for the term “shingles vaccine” and 1.5-fold increase in searches for “zoster vaccine.”

### Findings for Other Herpes Zoster-Related Terms

**Figure 4:**
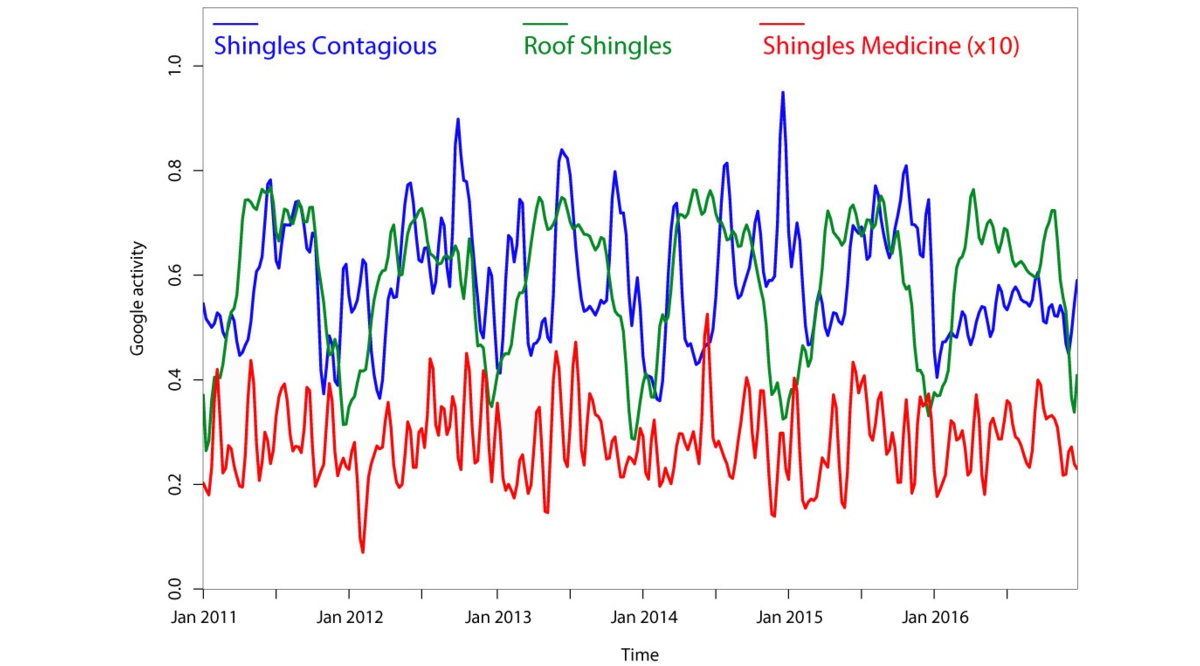
Smoothed Google searches for “shingles contagious,” “roof shingles,” and “shingles medicine” from 2011-2017, normalized as relative search volume (0-1.0). Searches for “roof shingles” are shown to illustrate seasonality of an unrelated search for “shingles.” Smoothing was conducted as in Figure 1.

**Figure 5:**
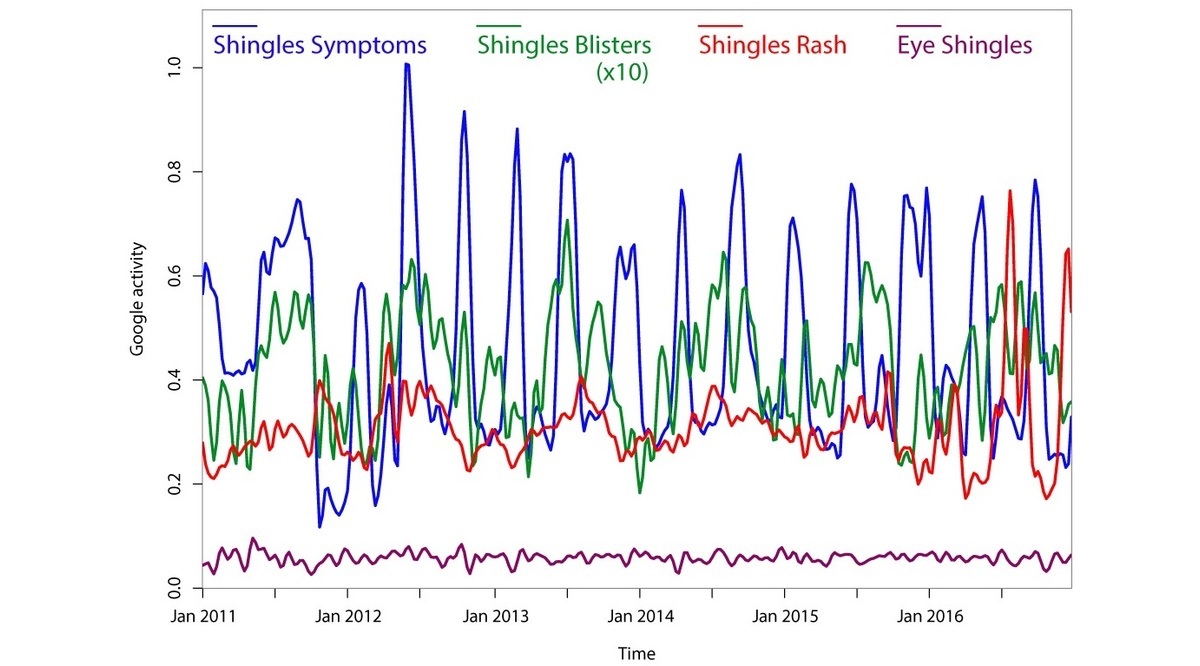
Smoothed Google searches for “shingles symptoms,” “shingles blisters,” “shingles rash,” and “eye shingles” from 2011-2017, normalized as relative search volume (0-1.0).

While this study primarily focused on trends in HZ vaccination, we also used the same techniques to explore trends for other HZ-related terms that are less directly associated with vaccination, including “shingles contagious ” and “shingles medicine” (Figure 4); “shingles symptoms,” “shingles blisters,” “shingles rash,” and “eye shingles” (Figure 5); and “skin rash,” “shingles itch,” “shingles pain,” and “shingles treatment” (data not shown). Figure 4 also illustrates trends in searches for the possible covariate “roof shingles” that also exhibits an annual periodicity but has a slightly shifted peak season compared to the terms “shingles contagious” and “shingles medicine” (*P*<.001). Although we did not find sharp increases in searches for these other HZ-related terms, we did find a periodicity for many of them. The terms “shingles blisters,” “shingles itch,” “shingles medicine,” “skin rash,” and “shingles rash” showed a statistically significant annual trend (see Table 1). The terms “shingles contagious,” “eye shingles,” and “shingles pain” showed no evidence of periodicity. The term “shingles symptoms” showed a highly significant periodicity with a 20-week cycle *(P*<.001). The term “shingles treatment” showed a similar 20-week periodicity that did not reach significance *(P*=.11) according to Fisher’s g test (although Morlet wavelet decomposition revealed a highly significant periodicity which began in early 2012).

**Table 1:** Periodicity of searches for herpes zoster-related terms.

Search Term	Frequency (cycles per year)	Peak Season(s)	Fisher’s g
Shingles Blisters	1	Summer	<.001
Shingles Itch	1	Summer	<.001
Shingles Rash	1	Summer	<.001
Skin Rash	1	Summer	<.001
Shingles Medicine	1	Summer	<.001
Eye Shingles	1	N/A^a^	.88
Shingles Contagious	<1	N/A	<.001
Shingles Pain	<1	N/A	.01
Shingles Treatment	2.6	N/A	.11
Shingles Symptoms	2.6	N/A	<.001

^a^N/A: not applicable.

## Discussion

### Principal Findings

Our current analysis of historic changes and seasonal patterns of public search interest over time related to the HZ vaccine identified a highly significant annual trend for searches related to vaccination, which appeared in part to be related to utilization of Medicare benefits, covaccination with the influenza vaccine, and advertising campaigns. This finding provides us with a better understanding of public interest in the HZ vaccine, which can be applied to informing future efforts to increase vaccine coverage.

Our results also suggest that public interest in a vaccine may be significantly affected by recommendations from health-related governmental departments. In the United States, United Kingdom, and Canada, a spike in search interest occurred in each country for vaccine-related terms soon after a recommendation by a health-related governmental organization was broadcast. With this in mind, it may be helpful to increase vaccine production or clinical stocks in anticipation of the increase in vaccine interest caused by these announcements. This factor is particularly important given the recent release of the new recombinant subunit vaccine. When the live attenuated vaccine was first released, there was some delay in vaccination efforts due to an insufficient supply of the vaccine [13]. The recent release of the recombinant subunit vaccine should not fall into the same trap, and vaccine availability could be increased accordingly to anticipate the likely increase in interest after any FDA or CDC announcements.

Finally, we report on a possible seasonality for interest in HZ as measured by an increase in Google search intensity in the summer. While trends in Google searches could be representative of trends in HZ incidence, there have been mixed reports about whether or not HZ is seasonal. Many studies have been published on the topic, ranging from reports of a single practice to regional health registries [47-58]. Six of these studies found that HZ exhibits a statistically significant seasonality, while two found that HZ incidence varies by season, but this trend was either not significant or its significance was not tested. All of the studies that reported a seasonal trend found an increase of HZ cases in the summer, which could explain the increase in interest for HZ vaccination in the summer. However, Google searches are only representative of interest in a topic and do not necessarily mean that a person has the disease they have searched for. Since the seasonality of HZ has been inconsistently characterized, we believe that this trend should be evaluated further utilizing a large health-specific dataset.

### Limitations

The use of social media and Internet search data to assess health matters has become more useful in recent years, but its methods come with limitations including the following for our current study. Google API data are reported as a normalized relative volume and can over-represent fluctuations in searches for terms with low post volumes. Second, Google Searches also estimate interest in a given topic, but not the follow through of getting vaccinated. Likewise, the periodicity of searches for HZ vaccination may not correspond to trends in actual utilization of the vaccine. As mentioned previously, our findings could benefit from validation using large administrative clinical datasets.

### Conclusions

Google searches about HZ vaccination exhibit an annual periodicity in the fall and significant peaks after recommendations from large public health organizations are broadcast. Given previous issues with HZ vaccine availability and coverage, these trends may help predict when to increase supply of the new recombinant subunit vaccine to meet changes in demand, and when to increase awareness to improve coverage.

## Abbreviations

API: application programming interface
FDA: United States Food and Drug Administration
HZ: herpes zoster
NACI: Canadian National Advisory Committee on Immunization
NIH-NEI: National Institutes of Health National Eye Institute
